# Heterocycles 36. Single-Walled Carbon Nanotubes-Bound *N*,*N*-Diethyl Ethanolamine as Mild and Efficient Racemisation Agent in the Enzymatic DKR of 2-Arylthiazol-4-yl-alanines

**DOI:** 10.3390/molecules21010025

**Published:** 2015-12-25

**Authors:** Denisa Leonte, László Csaba Bencze, Csaba Paizs, Monica Ioana Toşa, Valentin Zaharia, Florin Dan Irimie

**Affiliations:** 1 Department of Organic Chemistry, ”Iuliu Haţieganu” University of Medicine and Pharmacy, RO-400012 Cluj-Napoca, Victor Babeş 41, Romania; hapau.denisa@umfcluj.ro; 2 Biocatalysis and Biotransformation Research Group, Babeş-Bolyai University, RO-400028 Cluj-Napoca, Arany János 11, Romania; cslbencze@chem.ubbcluj.ro (L.C.B.); paizs@chem.ubbcluj.ro (C.P.); mtosa@chem.ubbcluj.ro (M.I.T.)

**Keywords:** hydrolases, dynamic kinetic resolution, racemisation agent, l-α-amino acids, thiazole

## Abstract

In this paper we describe the chemoenzymatic synthesis of enantiopure l-2-arylthiazol-4-yl alanines starting from their racemic *N*-acetyl derivatives; by combining the lipase-catalysed dynamic kinetic resolution of oxazol-5(4*H*)-ones with a chemical and an enzymatic enantioselective hydrolytic step affording the desired products in good yields (74%–78%) and high enantiopurities (*ee* > 99%). The developed procedure exploits the utility of the single-walled carbon nanotubes-bound diethylaminoethanol as mild and efficient racemisation agent for the dynamic kinetic resolution of the corresponding oxazolones.

## 1. Introduction

Optically-active α-amino acids bearing heterocyclic side chains are of great utility in various fields, not only individually, but especially incorporated in more complex structures, such as peptides and proteins, for the creation of new peptide-based pharmaceutical drug candidates [1,2]. The thiazole core frequently appears in many natural peptides, such as the Bleomycin family (anti-cancer glycopeptide antibiotics) [3], Nocathiacins [4], Aeruginazoles [5], and Thiazomycins [6] (a new class of cyclic thiopeptide antibiotics). The biological potential of this heterocyclic ring system is actually exploited for the design of new thiazole-bearing biologically active compounds, many of them being introduced in therapy. Enantiopure l-α-2-arylthiazole-4-yl alanines constitute chiral synthons with potential applications in drug design, especially when an extended conjugation is beneficial for interaction with pharmacological receptors. For example, the synthesis of new melanotropin analogues incorporating l-α-2-arylthiazole-4-yl alanines has recently been reported [2].

Lipases are often used as biocatalysts for the stereoselective production of variously functionalized optically-active products, due to their ability to transform a wide range of unnatural substrates in a regio- and stereoselective manner, not only in hydrolysis, but also in alcoholysis, aminolysis, or hydrazinolysis reactions using esters, lactones, or lactames as substrates [7].

Despite the success of enzyme-catalysed kinetic resolutions (KR) for the synthesis of a wide range of chiral building blocks, the increasing demand to develop transformations that are not limited by a maximum yield of only 50% drives the development of dynamic kinetic resolution (DKR) processes [8] in which the unreactive enantiomer equilibrates *in situ* under the reaction conditions with the most reactive antipode. Thus, DKR reactions provide the products in theoretical quantitative yields, with high enantiomeric excesses.

**Scheme 1:**
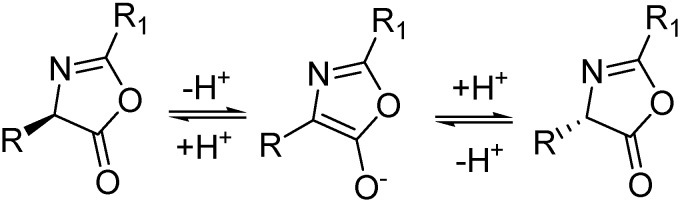
Base-catalysed racemization of oxazolones [12].

The enzymatic DKR of oxazolones was successfully employed for the synthesis of various alanine derivatives [9,10,11,12,13,14]. The oxazolones, due to the low p*Ka* of the C-4 proton and their inherent reactivity towards lipase-catalysed alcoholysis [9], are excellent substrates for the DKR reaction (Scheme 1). For an efficient DKR, one important requirement is related to the racemisation of the less-reactive enantiomer, which must be rapid under the reaction conditions, and the racemizing agent should not catalyse non-enzymatic secondary reactions, which could decrease the enantiopurity of the desired product.

If the spontaneous racemisation is faster than the enzymatic alcoholysis, there is no need to use racemisation agents as in the case of the chemoenzymatic procedures developed for the synthesis of benzofuranyl and benzotiophenyl alanines [13]. However, in case of the recently-reported DKR of phenylfuranyl derivatives, the enzymatic reactions showed higher velocity than the substrate racemisation, forcing the use of triethylamine as racemisation agent, which decreased the enzyme selectivity [14]. In order to alleviate the selectivity decrease caused by the racemisation agent, herein we describe the use of single-walled carbon nanotubes (SWCNT)-bound diethylaminoethanol in the lipase-catalysed dynamic kinetic resolution of the arylthiazole-based oxazolones. The covalent binding of the *N*,*N*-diethylaminoethanol on carboxy-functionalized SNWCNT_COOH_ was performed using glycerol diglycidyl ether as cross-linker, according to the procedure developed for the immobilization of *Pc*PAL [15] and Lipase B from *Candida antarctica* (CaL-B) [16], the remaining free tertiary amine functionality serving as organic base for the racemization process (Scheme 1). The developed chemoenzymatic procedure for the synthesis of l-2-arylthiazol-4-yl alanines involves two stereoselective enzymatic steps: the lipase-catalysed DKR of the corresponding 4-((2-arylthiazol-4-yl)methyl)-2-methyloxazol-5(4*H*)-ones, followed by Acylase I-mediated hydrolysis.

## 2. Results and Discussion

### 2.1. Synthesis of Racemic Substrates

**Scheme 2:**
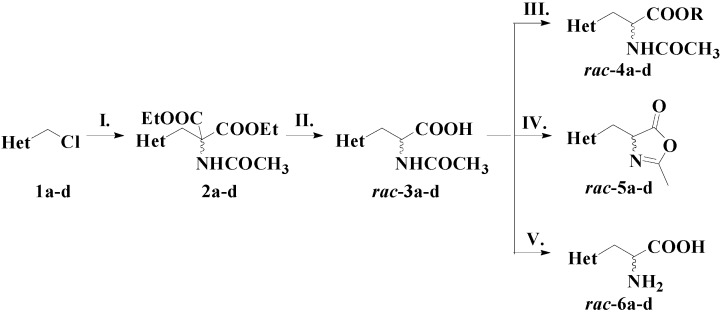
Synthesis of racemic 2-arylthiazol-4-yl alanines and derivatives. Reagents and conditions: **I**. NaH, CH_3_CONHCH(COOEt)_2_/DMF, 60 °C; **II**. (a). Hydrolysis of the ester groups: 10% KOH, reflux, 4 h; (b). Decarboxylation: toluene, reflux, 2 h; **III**. Alcohol (MeOH, EtOH, *n*-PrOH, *n*-BuOH), CDI/THF; **IV**. DCC/CH_2_Cl_2_; **V**. 18% HCl, reflux, 4 h.

The synthesis of racemic 2-arylthiazol-4-yl alanines *rac*-**6a**–**d** and their derivatives *rac*-**3-5a**–**d** is depicted in Scheme 2. 2-Aryl-4-chloromethylthiazoles **1a**–**d** were synthesized through the Hantzsch condensation of the corresponding thioamides with 1,3-dichloroacetone [17]. 2-Acetamido-3-(2-arylthiazol-4-yl)propanoic acids *rac*-**3a**–**d** were obtained according to the general malonic ester synthesis [13], starting from the halogenated derivatives **1a**–**d** through a coupling step with diethylacetamidomalonate, followed by a basic hydrolysis and a decarboxylation reaction.

The racemic esters *rac*-**4a**–**d** were obtained by treatment of *rac*-**3a**–**d** with different alcohols (methanol, ethanol, *n*-propanol, *n*-butanol) in the presence of carbonyldiimidazole (CDI).

The cyclisation of *rac*-**3a**–**d** in the presence of *N*,*N*′-dicyclohexylcarbodiimide (DCC), in dry dichloromethane, afforded the corresponding oxazol-5(4*H*)-ones *rac*-**5a**–**d**.

The racemic 2-arylthiazol-4-yl alanines *rac*-**6a**–**d** were obtained by acidic hydrolysis of the corresponding *N*-acetyl derivatives *rac*-**3a**–**d**.

### 2.2. Chemoenzymatic Synthesis of l-2-Arylthiazol-4-yl Alanines

**Scheme 3:**
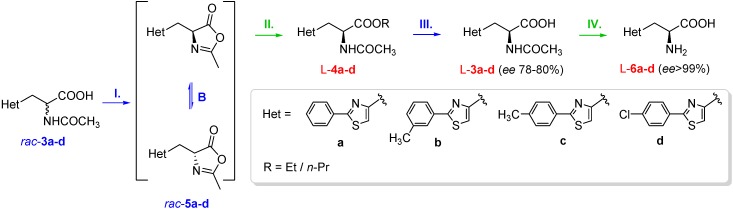
Stereoselective chemoenzymatic synthesis of l-2-arylthiazol-4-yl alanines and their derivatives (preparative scale). Reagents and conditions: **I.** DCC/CH_2_Cl_2_, 0 °C; **II.** CaL-B, ethanol (for dynamic kinetic resolution, DKR, of *rac*-**5a**–**c**)/*n*-Propanol (for DKR of *rac*-**5d**), acetonitrile; **III.** Na_2_CO_3_, H_2_O, reflux; **IV.** Acylase I, pH 7–8.

Racemic 2-acetamido-3-(2-arylthiazol-4-yl)propanoic acids *rac*-**3a**–**d** were used as starting materials for the stereoselective chemoenzymatic synthesis of l-2-arylthiazol-4-yl alanines (Scheme 3). The racemic oxazol-5(4*H*)-ones *rac*-**5a**–**d** obtained through cyclisation were used as substrates in the lipase-catalysed DKR process, with various alcohols as nucleophiles. The resulting l-2-acetamido-3-(2-arylthiazol-4-yl)propanoic esters l-**4a**–**d** were chemically hydrolysed under mild basic conditions ensured by Na_2_CO_3_. The obtained l-2-acetamido-3-(2-arylthiazol-4-yl)propanoic acids l-**3a**–**d** were converted into the corresponding l-2-arylthiazol-4-yl alanines l-**6a**–**d** by enantioselective hydrolysis of the amide bond, catalysed by Acylase I.

**Figure 1:**
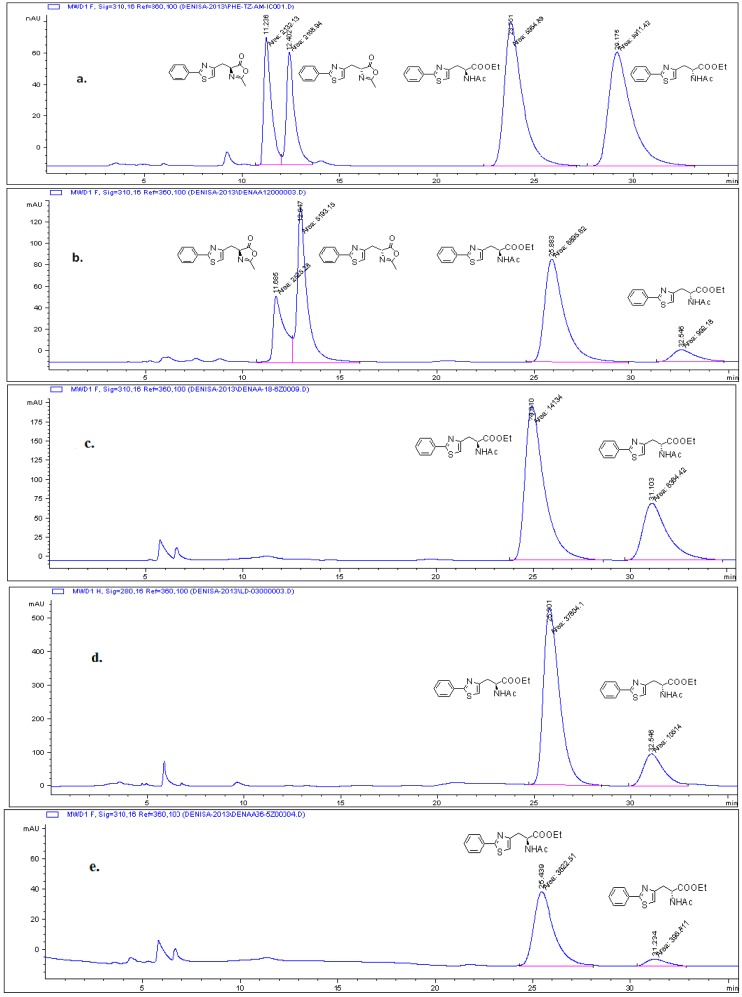
(**a**) Elution diagram of the mixture of the racemic starting material *rac*-**5a** and racemic product *rac*-**4a** for the lipase-catalysed DKR; (**b**) Elution diagram of the CaL-B catalysed DKR of oxazolone *rac*-**5a** in acetonitrile, using ethanol as nucleophile, without racemisation catalyst after 24 h and (**c**) after total consumption of the substrate (6 days); (**d**) Elution diagram of the CaL-B catalysed DKR of oxazolone *rac*-**5a** with ethanol in acetonitrile, using 0.25 eq. triethylamine as racemisation catalyst after total consumption of the substrate (24 h); (**e**) Elution diagram of the CaL-B catalysed DKR of racemic oxazolone *rac*-**5a** with ethanol, in acetonitrile, in the presence of the racemisation catalyst *N*,*N*-diethylaminoethanol immobilized on SWNCT, after total consumption of the substrate (36 h). HPLC method: Chiralpak IC, *n*-hexane: 2-propanol 80:20 *v*/*v*, UV-detection.

In order to investigate the stereoselectivity of the enzymatic processes, first the chiral HPLC separation of *rac*-**3-6a**–**d** was established (Figure 1a, see Section 3.1). Further, the enzymatic DKR of oxazol-5(4*H*)-ones *rac*-**5a**–**d** was studied through the screening of the reaction conditions (enzyme, nucleophile, solvent, racemisation catalyst) using the unsubstituted compound *rac*-**5a** as model substrate in order to obtain the highest enantiopurities. Therefore, we first tested the alcoholysis of *rac*-**5a** in the presence of various lipases in neat alcohol. Among the tested lipases, only two showed promising results (Table 1): Lipozyme *Mucor miehei* gave poor stereoselectivity (44% *ee*, Table 1, entry 7), while CaL-B (Novozyme 435) showed a higher stereoselectivity (72% *ee*, Table 1, entry 4), using ethanol as nucleophile. Consequently, CaL-B was chosen for further DKR studies.

**Table 1:** Lipase catalysed kinetic resolution of *rac*-**5a** in ethanol, after 4.5 h reaction time.

Entry	Lipase	*c %*	*ee_p_* (%)
1	*Candida rugosa* lipase	10	<2
2	Lipase AK “Amano”	92	17 *
3	*Burkholderia cepacia* lipase	14	11 *
4	*Candida antarctica* lipase B	70	72
5	*Candida cylindraceae lipase*	9	<2
6	Lipase F	10	9
7	Lipozyme *Mucor miehei*	95	44

* inverse selectivity.

It is known that the nature of the nucleophile and of the solvent could significantly influence the stereoselectivity of the enzymatic reaction, therefore the CaL-B mediated ring opening of *rac*-**5a** was performed in the presence of various alcohols (methanol, ethanol, *n*-propanol, and *n*-butanol) as nucleophiles (Table 2). Ethanol as nucleophile (52% *ee*, Table 2, entry 2) provided the highest selectivity value, therefore further screening was performed with ethanol as nucleophile (Table 3).

**Table 2:** The CaL-B mediated ring opening of oxazolone *rac*-**5a** using various alcohols as nucleophiles, after complete conversion of substrates.

Entry	Alcohol	Product	*ee %*
1	Methanol	l-**4a** methyl ester	8 ^a^
2	Ethanol	l-**4a** ethyl ester	52 ^b^
3	*n*-Propanol	l-**4a** *n*-propyl ester	47 ^a^
4	*n*-Butanol	l-**4a** *n*-butyl ester	35 ^a^

^a^ after total conversion in 8 h; ^b^ after total conversion in 24 h.

**Table 3:** Solvent screening for the enantioselective alcoholysis of oxazolone *rac*-**5a**, with CaL-B and ethanol, after total consumption of the substrate (6 days).

Entry	Solvent	*ee_p_* %
1	1,4-Dioxane	51.3
2	Dichloromethane ^1^	-
3	Toluene	58.9
4	Acetonitrile	37.3
5	Tetrahydrofurane	29.2
6	Diethylether	30.0

^1^ low conversion (<2%).

The solvent screening showed that higher selectivities and longer reaction times were obtained when compared with the reactions performed in neat ethanol. Furthermore, the observed decrease of the enantiopurities of the produced l-**4a** with increasing conversions (Figure 1b,c) indicates a similar behaviour of the DKR as those reported for the phenylfuran-2-yl derivatives [14], when the racemisation of oxazolone enantiomers was slower than enzymatic alcoholysis. Therefore, the lowered reaction rate of enzymatic alcoholysis from the solvent screening can be beneficial, supported by the increased enantiomeric excess of the product (Table 3, entry 3).

**Scheme 4:**
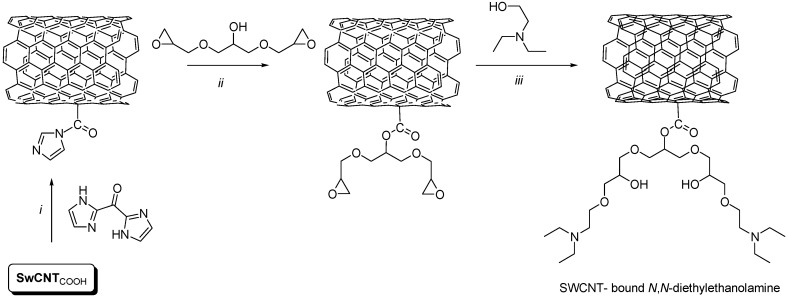
Immobilisation of *N,N*-diethylaminoethanol on carboxy-functionalized single-walled carbon nanotubes (SWCNT_COOH_). Reaction conditions: **i.** 1,1′-carbonyldiimidazole, anhydrous dichloromethane, r.t.; **ii.** glycerol diglycidyl ether, anhydrous dichloromethane, 24 h, r.t.; **iii.** *N*,*N*-diethylaminoethanol, anhydrous dichloromethane, 24 h, r.t.

Additionally, in order to increase the racemisation process, the use of organic bases (triethylamine, pryidine, *etc.*) proved successful in several cases [10,14], however their amount in the reaction media must be carefully controlled in order to avoid the decrease in the selectivity and activity of the enzyme [14]. When performing the racemisation in the presence of different low, catalytic amounts (0.5, 0.25, 0.1 eq.) of organic base, the DKR reached total conversions more rapidly (1 day instead of 6 days for DKR process without racemisation catalysts), however the selectivities still remained unsatisfactory (*ee* < 56%, Figure 1d). The blank reaction performed without enzyme proved that even small amounts of weak bases are catalysing the chemical alcoholysis of the oxazolone *rac*-**5a**, resulting in lowered enantiomeric excess of the l-**4a**. In order to avoid the chemical alcoholysis of the substrates and the free entrance of the reactive organic base into the catalytic site of the enzyme, which might also be responsible for the decrease of selectivity, we decided to develop an immobilized racemisation catalyst with large hydrophobic surface area and low diffusional resistance. The developed catalyst would allow for the fast racemisation of the substrate, but would be unable to enter and interact directly with the catalytic site of the enzyme. Thus, catalytic amounts (0.5, 0.25, 0.1 eq.) of diethylaminoethanol covalently bound to carboxy-functionalized single-walled carbon nanotubes (SWCNT_COOH_) (Scheme 4) were tested in the CaL-B catalysed DKR of *rac*-**5a** in various solvents, using ethanol as nucleophile. Accordingly, for the enzymatic DKR of *rac*-**5a**, the enantiomeric excess of the obtained l-**4a** product considerably increased, proving the beneficial effects of the immobilized racemisation catalyst, which is maintaining the substrate in racemic form during the reaction (Figure 1e). The optimal conditions were found to be acetonitrile as solvent (Table 4, entry 4) and ethanol as nucleophile (Table 5, entry 2). Moreover, in the presence of the nanotube-supported base, no chemical ethanolysis was detected in enzyme-less blank experiments, which can be explained by the high affinity of the hydrophobic oxazolones to the surface of the carbon nanotube, which at the same time keeps away the polar nucleophile, avoiding chemical ethanolysis.

In order to increase the enantioselectivity of the DKR process, different ratios between lipase, substrate, racemisation catalyst, and ethanol were tested. Two different amounts of CaL-B were used (5 mg and 10 mg of lipase for 10 mg of substrate), in the presence of different amounts of immobilized racemisation catalyst (3 mg and 6 mg) and ethanol (3 eq. and 6 eq.) in acetonitrile at room temperature. The enantiomeric excesses of the DKR products remained in the interval 70%–80%. The best results (80% *ee*) were obtained with 10 mg CaL-B and 6 mg of racemisation catalyst for 10 mg of substrate, with 3 eq. of ethanol.

**Table 4:** Solvent screening for the enantioselective alcoholysis of *rac*-**5a** with CaL-B and ethanol, in the presence of racemisation catalyst (diethylaminoethanol immobilized on SWCNT) (reaction time: 44 h).

Entry	Solvent	*c %*	*ee_p_* %
1	1,4-Dioxane	91	61
2	Dichloromethane	<2	<2
3	Toluene	>99	61
4	Acetonitrile	>99	80
5	Tetrahydrofurane	60	64
6	Diethylether	>99	30

By performing the DKR of *rac*-**5a** at 30 °C, 40 °C, and 50 °C, an increase of the reaction rate was observed, however no increase of the enantioselectivity was observed; moreover, at 50 °C, the *ee* of the product decreased to 70%.

Having in hand the optimized reaction conditions of the enzymatic DKR of *rac*-**5a**, we decided to extend the same DKR procedure for the other thiazole-based oxazolones *rac*-**5b**–**d**. Similar good results were obtained when *rac*-**5b**,**c** were used as substrates, with ethanol (3 eq.) and acetonitrile as solvent (Table 5, entries 5, 6), while in the case of *rac*-**5d**, the obtained lower enantioselectivities forced us to retake the nucleophile screening. Finally, the use of *n*-propanol as nucleophile provided the best results (Table 5, entry 7).

**Table 5:** CaL-B mediated DKR of *rac*-**5a**–**d**, in acetonitrile, with different alcohols, in the presence of 0.25 eq. immobilized diethylaminoethanol as racemisation catalyst, at total conversion of the substrate (reaction time: 44 h).

Entry	Substrate	Alcohol	Product	*ee_p_* %
1	*rac*-**5a**	Methanol	l-**4a** methyl ester	71
2	*rac*-**5a**	Ethanol	l-**4a** ethyl ester	80
3	*rac*-**5a**	*n*-Propanol	l-**4a** *n*-propyl ester	76
4	*rac*-**5a**	*n-*Butanol	l-**4a** *n*-butyl ester	58
5	*rac*-**5b**	Ethanol	l-**4b** ethyl ester	78
6	*rac*-**5c**	Ethanol	l-**4c** ethyl ester	78
7	*rac*-**5d**	*n*-Propanol	l-**4d** *n*-propyl ester	80

Using the optimal conditions found for the small scale reactions, the preparative scale enzymatic DKR of oxazolones *rac*-**5a**–**d** was performed at room temperature, affording the corresponding l-2-acetamido-3-(2-arylthiazol-4-yl)propanoic esters l-**4a**–**d** in excellent yields (>93%) and moderate enantiomeric excesses (78%–80%, Table 6). The total conversions of the preparative scale DKR processes were achieved after 2 days, in the case of *rac*-**5a**–**c**, and respectively after 3 days when *rac*-**5d** was used as substrate.

Further, the enantiomerically-enriched DKR products l-**4a**–**d** were hydrolysed under mild basic conditions, with good yields (>98%) to l-**3a**–**d**, without altering the *ee* (Table 6, entry 2). In order to increase the *ee* of the final products in enantiopure form, the formed *N*-acetyl amino acids l-**3a**–**d** were submitted to the Acylase I-catalysed enantioselective hydrolysis of the amide group (Scheme 2), affording the corresponding amino acids l-**6a**–**d** with excellent enantiopurity and good global yields (Table 7).

The expected L-configuration of the obtained enantiopure 2-arylthiazole-4-yl alanines **6a**–**d** was confirmed by measuring their specific rotation, which were consistent with the literature values [18].

**Table 6:** Yields and *ee* values for each step of the preparative scale chemoenzymatic synthesis of l-2-arylthiazole-4-yl alanines l-**6a**–**d**.

Entry	Enzymatic/Chemical Step of the Preparative Scale Synthesis	Substrate	Product	Yield %	*ee_p_* %
1	CaL-B catalysed DKR of oxazolones *rac*-**5a**–**d** with ethanol/*n*-propanol (3 eq.) and immobilized diethylaminoethanol, in acetonitrile	*rac*-**5a**	l-**4a** ethyl ester	95	80
		*rac*-**5b**	l-**4b** ethyl ester	96	78
		*rac*-**5c**	l-**4c** ethyl ester	93	78
		*rac*-**5d**	l-**4d** *n*-propyl ester	95	80
2	Chemical hydrolysis of the *N*-acetyl amino esters l-**4a**–**d** under mild basic conditions (Na_2_CO_3_/H_2_O)	l-**4a** ethyl ester	l-**3a**	99	80
		l-**4b** ethyl ester	l-**3b**	98	78
		l-**4c** ethyl ester	l-**3c**	99	78
		l-**4d** *n*-propyl ester	l-**3d**	99	80
3	Acylase I-catalysed enantioselective hydrolysis of the *N*-acetyl amino acids l-**3a**–**d**	l-**3a**	l-**6a**	92	>99
		l-**3b**	**l**-**6b**	92	>99
		l-**3c**	l-**6c**	91	>99
		l-**3d**	l-**6d**	92	>99

**Table 7:** Global yields and specific rotations for enantiopure l-2-arylthiazole-4-yl alanines l-**6a**–**d**, obtained by CaL-B mediated DKR of *rac*-**5a**–**d** followed by Acylase I-mediated enantioselective hydrolysis of l-**3a**–**d**.

Entry	Product	Global Yield ^a^ (%)	*ee* (%)	${\left[\alpha \right]}_{\text{D}}^{28}$
1	l-**6a**	78	>99	−0.20 ^b^
2	l-**6b**	77	>99	−0.26 ^b^
3	l-**6c**	74	>99	−0.27 ^b^
4	l-**6d**	78	>99	−0.35 ^b^

^a^ calculated based on the starting material *rac*-**3a**–**d**; ^b^ (CH_3_COOH, *c* = 5 mg/mL).

## 3. Experimental Section

### 3.1. Analytical Methods

The ^1^H-NMR and ^13^C-NMR spectra were recorded on a Bruker Avance DPX-300 spectrometer (Bruker, Billerica, MA, USA) operating at 600 and 150 MHz, respectively. Chemical shifts on the δ scale are expressed in ppm values from tetramethylsilane as internal standard. ESI^+^ MS spectra were recorded on a GC-MS Shimadzu QP 2010 Plus spectrometer (Shimadzu Europa GmbH, Duisburg, Germany) using direct injection, at 30–70 eV.

High performance liquid chromatography analyses were conducted with an Agilent 1200 instrument (Agilent Technologies, Santa Clara, CA, USA), using a Chiralpak IC column (4.6 × 250 mm, Daicel Chiral Technologies Europe, Essex, UK) and a mixture of *n*-hexane and 2-propanol 80:20 (*v*/*v*) as eluent for the enantiomeric separation of *rac*-**4a**–**d**, an Astec Chirobiotic V2 column, and a mixture of methanol, acetic acid, triethylamine (TEA) 200:0.15:0.15 (*v*/*v*/*v*) as eluent for the enantiomeric separation of *rac*-**3a**–**d**, and a Chiralpak Zwix(+) column with a mixture of methanol (50 mM formic acid, 25 mM diethylamine (DEA)), acetonitrile, water 49:49:2 (*v*/*v*/*v*), for the chiral separation of *rac*-**6a**–**d**, all at 1 mL/min flow rate. The gradient separation method and the retention times of the enantiomers are shown in Table 8.

Thin layer chromatography (TLC) was carried out using Merck Kieselgel 60F_254_ sheets (Merck, Darmstadt, Germany). Spots were visualized in UV light or by treatment with 5% ethanolic ninhydrin solution and heating of the dried plates. Preparative chromatographic separations were performed using column chromatography on Merck Kieselgel 60 Å (63–200 μm). Optical rotations were determined on a Bellingham-Stanley ADP 220 polarimeter using acetic acid as solvent.

**Table 8:** The retention times for the enantiomers of *rac-***3-6a**–**d**.

**Separation Conditions**	RP-HPLC Astec chirobiotic V2 column, eluent: MeOH:CH_3_COOH:Et_3_N 200:0.15:0.15 *v*/*v*/*v*							
**Compound**	l-**3a**	d-**3a**	l-**3b**	d-**3b**	l-**3c**	d-**3c**	l-**3d**	d-**3d**
**R_t_ (min)**	4.4	6.1	4.5	6.0	4.8	6.4	5.5	7.0
**Separation Conditions**	HPLC Chiralpak IC, eluent: *n*-hexane:2-propanol 80:20 *v*/*v*							
**Compound**	(*S*)-**5a**	(*R*)-**5a**	(*S*)-**5b**	(*R*)-**5b**	(*S*)-**5c**	(*R*)-**5c**	(*S*)-**5d**	(*R*)-**5d**
**R_t_ (min)**	11.2	12.4	11.5	12.6	11.3	12.5	10.2	12.0
**Compound**	l-**4a**	d-**4a**	l-**4b**	d-**4b**	l-**4c**	d-**4c**	l-**4d**	d-**4d**
**R_t_ (min)**	29.9 ^a^	36.4 ^a^	25.0 ^b^	29.2 ^b^	28.5 ^b^	34.0 ^b^	28.9 ^a^	35.5 ^a^
	24.4 ^b^	30.2 ^b^					25.0 ^b^	30.0 ^b^
	20.3 ^c^	25.2 ^c^					19.0 ^c^	23.2 ^c^
	19.7 ^d^	24.0 ^d^					18.6 ^d^	22.7 ^d^
**Separation Conditions**	RP-HPLC Chiralpak Zwix(+), eluent: MeOH (50 mM HCOOH, 25 mM diethylamine):acetonitrile:water 49:49:2 *v*/*v*/*v*							
**Compound**	*rac*-**3a**		*rac*-**3b**		*rac*-**3c**		*rac*-**3d**	
**R_t_ (min)**	5.9		4.2		5.2		4.1	
**Compound**	l-**6a**	d-**6a**	l-**6b**	d-**6b**	l-**6c**	d-**6c**	l-**6d**	d-**6d**
**R_t_ (min)**	10.9	18.8	9.5	20.2	10.3	21.2	11.3	24.9

^a^ methyl ester; ^b^ ethyl ester; ^c^ *n*-propyl ester; ^d^ *n*-butyl ester.

Melting points were determined on open glass capillaries using an Electrothermal IA 9000 digital apparatus.

### 3.2. Reagents and Solvents

The commercial chemicals and solvents were products of Sigma Aldrich (Sigma Aldrich Chemie Gmbh, Steinheim, Germany) or Fluka (Buchs, Switzerland). All solvents were purified and dried by standard methods as required. Carboxy-functionalized Single walled carbon nanotubes (SWCNT_COOH_) were purchased from Organic Chemicals Co. Ltd. (Chengdu, China). Lipase B from *Candida antarctica* (CaL-B, Novozym 435) was purchased from Novozymes, Bagsvaerdt, Denmark. Lipases from *Candida rugosa* (CrL), *Candida cylindracea* (CcL), *Mucor miehei* (MmL) and Acylase I were purchased from Fluka. Lipases from *Pseudomonas fluorescens* (AK free), *Burkholderia cepacia* (BcL), and lipase F were from Amano, Chipping Norton, UK.

### 3.3. Chemical Synthesis of Racemic 2-Arylthiazol-4-yl Alanines and Their Derivatives

#### 3.3.1. Synthesis of Racemic 2-Acetamido-3-(2-arylthiazol-4-yl)propanoic Acids *rac*-**3a**–**d**

A dispersion of 60% NaH in mineral oil (0.84 g, 21 mmol) was suspended in dry *N*,*N*-dimethylformamide (12 mL) and stirred under argon at room temperature. After 30 min, diethyl acetamidomalonate (4.34 g, 20 mmol) was added, the mixture was stirred for 30 min and cooled, followed by the dropwise addition of the halogenated thiazole derivative **1a**–**d** (22 mmol) dissolved in dry *N*,*N*-dimethylformamide (5 mL). The reaction mixture was stirred for 3 h at room temperature, and for the next 4 h at 60 °C. The solution was cooled and poured on a water–ice mixture. The formed precipitate was filtered off, dried, and suspended in an aqueous solution of 10% KOH (4–5 mL). The reaction mixture was refluxed for 4 h, in order to hydrolyse the ester groups. The resulting solution was cooled and the pH was adjusted to 1 with concentrated HCl. The formed precipitate was filtered, dried, suspended in toluene (10 mL), and refluxed for 2 h, until complete decarboxylation. The formed white crystals of 2-acetamido-3-(2-arylthiazol-4-yl)propanoic acids were isolated by filtration and dried.

*2-Acetamido-3-(2-phenylthiazol-4-yl)propanoic acid* (*rac**-*****3a**): Yield: 64%; white solid; m.p. 175–176 °C; ^1^H-NMR (600 MHz, DMSO) δ 8.25 (1H, NH), 7.91 (dd, *J* = 7.9, 1.4 Hz, 2H), 7.52–7.46 (m, 3H), 7.38 (s, 1H), 4.60 (td, *J* = 8.7, 5.0 Hz, 1H), 3.14 (ddd, *J* = 23.7, 14.6, 7.0 Hz, 2H), 1.81 (s, 3H). ^13^C-NMR (151 MHz, DMSO) δ 172.99, 169.29, 166.38, 153.50, 133.11, 130.14, 129.21, 126.06, 116.24, 51.82, 32.89, 22.40; ESI-MS: 291.0800 (calculated: 291.0798, for C_14_H_14_N_2_O_3_S [M + H]^+^); *m*/*z* (%): 313 (24, [M + Na]^+^), 293 (4.5, [M + 3H]^+^), 292 (15.2, [M + 2H]^+^), 291 (100, [M + H]^+^), 284 (2.8), 279 (1.3), 273 (1.2).

*2-Acetamido-3-(2-m-tolylthiazol-4-yl)propanoic acid* (*rac**-*****3b**): Yield: 65%; white solid; m.p. 160–161 °C; ^1^H-NMR (600 MHz, DMSO) δ 7.97 (1H, NH), 7.73 (s, 1H), 7.68 (d, *J* = 7.7 Hz, 1H), 7.36 (t, *J* = 7.6 Hz, 1H), 7.30 (s, 1H), 7.26 (d, *J* = 7.4 Hz, 1H), 4.43 (m, 1H), 3.13 (ddd, *J* = 23.2, 14.6, 6.6 Hz, 2H), 2.37 (s, 3H), 1.79 (s, 3H). ^13^C-NMR (151 MHz, DMSO) δ 173.32, 168.80, 165.97, 154.70, 138.51, 133.24, 130.66, 129.08, 126.42, 123.28, 115.31, 52.99, 33.75, 22.61, 20.91; ESI^+^-MS: 305.0960 (calculated: 305.0954 for C_15_H_16_N_2_O_3_S [M + H]^+^); *m*/*z* (%): 327 (85.3, [M + Na]^+^), 307 (4.4, [M + 3H]^+^), 306 (16.5, [M + 2H]^+^), 305 (100, [M + H]^+^), 292 (3.0), 291 (21.2), 288 (4.2), 284 (1.7), 263 (30.4), 251 (21.8), 210 (4.8).

*2-Acetamido-3-(2-p-tolylthiazol-4-yl)propanoic acid* (*rac**-*****3c**): Yield: 67%; white solid; m.p. 181–182 °C; ^1^H-NMR (600 MHz, DMSO) δ 8.17 (1H, NH), 7.80 (d, *J* = 8.1 Hz, 2H), 7.31 (s, 1H), 7.30 (d, *J* = 8.0 Hz, 2H), 4.56 (dd, *J* = 13.2, 8.3 Hz, 1H), 3.12 (ddd, *J* = 23.6, 14.6, 6.9 Hz, 2H), 2.35 (s, 3H), 1.80 (s, 3H). ^13^C-NMR (151 MHz, DMSO) δ 173.03, 169.17, 166.41, 153.52, 139.88, 130.57, 129.72, 125.99, 115.50, 51.98, 33.03, 22.44, 20.93; ESI^+^-MS: 305.0967 (calculated: 305.0954, for C_15_H_16_N_2_O_3_S [M + H]^+^); *m*/*z* (%): 343 (100, [M + K]^+^), 327 (25.4, [M + Na]^+^), 307 (0.8, [M + 3H]^+^), 306 (3.2, [M + 2H]^+^), 305 (18.5, [M + H]^+^), 291 (0.9), 284 (1.8), 263 (1.5), 251 (1.3), 210 (0.2).

*2-Acetamido-3-(2-p-clorophenylthiazol-4-yl)propanoic acid* (*rac**-*****3d)**: Yield: 64%; white solid; m.p. 199–200 °C; ^1^H-NMR (600 MHz, DMSO) δ 8.20 (1H, NH), 7.92 (d, *J* = 7.2 Hz, 2H), 7.55 (d, *J* = 6.8 Hz, 2H), 7.40 (s, 1H), 4.57 (dd, *J* = 11.9, 8.9 Hz, 1H), 3.13 (ddd, *J* = 23.6, 14.6, 7.0 Hz, 2H), 1.80 (s, 3H). ^13^C-NMR (151 MHz, DMSO) δ 173.03, 169.29, 164.98, 153.89, 134.63, 131.98, 129.28, 127.77, 116.70, 51.99, 33.01, 22.46; ESI^+^-MS: 325.0408 (calculated: 325.0408, for C_14_H_13_ClN_2_O_3_S [M + H]^+^); *m*/*z* (%): 347 (12.5, [M + Na]^+^), 328 (11.1, [M + 2H]^+^, ^37^Cl), 327 (70.1, [M + H]^+^, ^37^Cl), 326 (3.2, [M + 2H]^+^, ^35^Cl), 325 (21.4, [M + H]^+^, ^35^Cl), 313 (9.5), 305 (32.6), 251 (18.7), 210 (3.7).

#### 3.3.2. Synthesis of Racemic 2-Acetamido-3-(2-arylthiazol-4-yl)propanoic Esters *rac*-**4a**–**d**

To a solution of racemic 2-acetamido-3-(2-arylthiazol-4-yl)propanoic acid *rac*-**3a**–**d** (0.5 mmol) and carbonyl diimidazole (90 mg, 0.55 mmol) in anhydrous THF (2 mL), ethanol (321 μL, 5.5 mmol) was added. The reaction mixture was stirred at room temperature overnight. The solvent was removed *in vacuo* and the crude product was purified with column chromatography on silica gel using dichloromethane:acetone 9:1 (*v*/*v*) as eluent. Methyl, *n*-propyl and *n*-butyl 2-acetamido-3-(2-arylthiazol-4-yl)propanoates were obtained by the same procedure, using methanol, propanol or butanol instead of ethanol.

*Ethyl 2-acetamido-3-(2-phenylthiazol-4-yl)propanoate* (*rac**-*****4a**): Yield: 64%; white solid; m.p. 116–117 °C; ^1^H-NMR (600 MHz, CDCl_3_) δ 7.94 (d, *J* = 4.0 Hz, 2H), 7.45–7.46 (m, 3H), 7.02 (s, 1H), 4.91–4.94 (m, 1H), 4.19 (q, *J* = 7.1 Hz, 2H), 3.37 (ddd, *J* = 17.4, 14.4, 3.7 Hz, 2H), 2.04 (s, 3H), 1.24 (t, *J* = 7.1 Hz, 3H). ^13^C-NMR (151 MHz, CDCl_3_) δ 171.32, 170.19, 168.65, 152.31, 133.18, 130.70, 129.25, 126.68, 115.93, 61.67, 52.16, 32.84, 23.42, 14.33; ESI^+^-MS: 319.1121 (calculated: 319.1111 for C_16_H_18_N_2_O_3_S [M + H]^+^); *m*/*z* (%): 357 (44.3, [M + K]^+^), 341 (16.5, [M + Na]^+^), 320 (18.8, [M + 2H]^+^), 319 (100, [M + H]^+^), 305 (10), 277 (14).

*Ethyl 2-acetamido-3-(2-m-tolylthiazol-4-yl)propanoate* (*rac**-*****4b**): Yield: 63%; yellow solid; m.p. 81 °C; ^1^H-NMR (600 MHz, CDCl3) δ 7.72 (s, 1H), 7.70 (d, *J* = 7.8 Hz, 1H), 7.32 (t, *J* = 7.6 Hz, 1H), 7.24 (d, *J* = 7.5 Hz, 1H), 6.97 (s, 1H), 4.92 (dd, *J* = 12.9, 5.2 Hz, 1H), 4.18 (q, *J* = 7.1 Hz, 2H), 3.32 (ddd, *J* = 46.0, 14.8, 5.2 Hz, 2H), 2.41 (s, 3H), 2.03 (s, 3H), 1.22 (t, *J* = 7.1 Hz, 3H). ^13^C-NMR (151 MHz, CDCl3) δ 171.40, 170.05, 168.60, 152.47, 138.88, 133.22, 131.18, 129.03, 127.12, 123.70, 115.61, 61.56, 52.12, 33.03, 23.36, 21.49, 14.27. ESI^+^-MS: 333.1270 (calculated: 333.1267 for C_17_H_20_N_2_O_3_S [M + H]^+^); *m*/*z* (%): 371 (61.0, [M + K]^+^), 355 (36.9, [M + Na]^+^), 334 (19.8, [M + 2H]^+^), 333 (100.0, [M + H]^+^), 319 (2.7), 305 (1), 291 (6.2).

*Ethyl 2-acetamido-3-(2-p-tolylthiazol-4-yl)propanoate* (*rac**-*****4c**): Yield: 65%; white solid; m.p. 98 °C; ^1^H-NMR (600 MHz, CDCl_3_) δ 7.78 (d, *J* = 8.0 Hz, 2H), 7.24 (d, *J* = 7.9 Hz, 2H), 6.94 (s, 1H), 4.91 (dt, *J* = 7.5, 5.1 Hz, 1H), 4.18 (q, *J* = 7.1 Hz, 2H), 3.31 (ddd, *J* = 50.6, 14.8, 5.0 Hz, 2H), 2.40 (s, 3H), 2.04 (s, 3H), 1.22 (t, *J* = 7.1 Hz, 3H). ^13^C-NMR (151 MHz, CDCl_3_) δ 171.45, 170.07, 168.56, 152.54, 140.60, 130.96, 129.81, 126.42, 115.22, 61.58, 52.15, 33.11, 23.43, 21.56, 14.31; ESI^+^-MS: 333.1265 (calculated: 333.1267 for C_17_H_20_N_2_O_3_S [M + H]^+^); *m*/*z* (%): 371 (12.1, [M + K]^+^), 355 (47.6, [M + Na]^+^), 334 (21.5, [M + 2H]^+^), 333 (100.0, [M + H]^+^), 319 (35), 305 (17.5), 291 (5), 259 (12.0), 253 (22.9), 217 (24.9).

*n-Propyl 2-acetamido-3-(2-p-chlorophenylthiazol-4-yl)propanoate* (*rac**-*****4d**): Yield: 64%; white solid; ^1^H-NMR (600 MHz, CDCl_3_) δ 7.86 (d, *J* = 8.5 Hz, 2H), 7.40 (d, *J* = 8.5 Hz, 2H), 6.97 (s, 1H), 4.94 (dt, *J* = 7.7, 5.1 Hz, 1H), 4.08 (t, *J* = 6.7 Hz, 2H), 2.02 (s, 3H), 1.73–1.65 (m, 2H), 0.89 (t, *J* = 7.4 Hz, 3H). ^13^C-NMR (151 MHz, CDCl_3_) δ 171.55, 170.34, 168.02, 153.77, 148.43, 131.36, 129.35, 127.67, 115.97, 67.24, 52.07, 34.06, 23.40, 21.70, 10.46; ESI^+^-MS: 367.0879 (calculated: 367.0878 for C_17_H_19_ClN_2_O_3_S [M + H]^+^); *m*/*z* (%): 405 ([M + K]^+^), 389 ([M + Na]^+^), 370 (6.6, [M + 2H]^+^, ^37^Cl), 369 (37.2, [M + H]^+^, ^37^Cl), 368 (19.5, [M + 2H]^+^, ^35^Cl), 367 (100, [M + H]^+^, ^35^Cl), 333 (10), 319 (1.8), 305 (1.7), 287 (1.5), 244 (1.8).

#### 3.3.3. Synthesis of Racemic 4-((2-Arylthiazol-4-yl)methyl)-2-methyloxazol-5(4*H*)-ones *rac*-**5a**–**d**

To a solution of racemic 2-acetamido-3-(2-arylthiazol-4-yl)propanoic acid *rac*-**3a**–**d** (1 mmol) in anhydrous dichloromethane (5 mL), a solution of *N*,*N*′-dicyclohexyl-carbodiimide (247.2 mg, 1.2 mmol) in anhydrous dichloromethane (2 mL) was added dropwise at 0 °C. The reaction mixture was stirred for 1 h at 0 °C. After the completion of the reaction (verified by TLC, eluent dichloromethane:acetone 9:1), the formed precipitate of dicyclohexyl urea was removed by filtration. The solvent was distilled off at reduced pressure, obtaining without further purifications the pure oxazol-5(4*H*)-ones *rac*-**5a**–**d**, which were directly used in the enzymatic reactions.

#### 3.3.4. Synthesis of Racemic 2-Arylthiazole-4-yl Alanines *rac*-**6a**–**d**

A suspension of racemic 2-acetamido-3-(2-arylthiazol-4-yl)propanoic acid *rac*-**3a**–**d** (50 mg) in 18% HCl (6 mL) was refluxed for 4 h. The solvent was removed by distillation at reduced pressure, affording the corresponding 2-arylthiazole-4-yl alanine *rac*-**6a**–**d** as hydrochloride salt, which was dried and washed several times with diethyl ether.

*2-Amino-3-(2-phenylthiazol-4-yl)propanoic acid* (*rac**-*****6a**): Yield: 91%; white powder; m.p. 241–248 °C for the hydrochloride salt, respectively m.p. over 300 °C with decomposition for the free amino acid; ^1^H-NMR (600 MHz, D_2_O) δ 7.82–7.79 (m, 2H), 7.62–7.38 (m, 4H), 4.44 (t, *J* = 6.5 Hz, 1H), 3.45 (ddd, *J* = 22.5, 15.6, 6.4 Hz, 2H). ^13^C-NMR (151 MHz, D_2_O) δ 172.4, 170.98, 170.67, 146.59, 132.00, 129.50, 126.90, 119.69, 52.24, 29.84; ESI^+^-MS: 249.0699 (calculated: 249.0692 for C_12_H_12_N_2_O_2_S [M + H]^+^); *m*/*z* (%): 263 (100), 249 (2.9, [M + H]^+^), 203 (1.5).

*2-Amino-3-(2-m-tolylthiazol-4-yl)propanoic acid* (*rac**-*****6b**): Yield: 88%; white powder; m.p. 225–238 °C for the hydrochloride salt, respectively m.p. 227–242 °C with decomposition for the free amino acid; ^1^H-NMR (600 MHz, D_2_O) δ 7.54 (m, 3H), 7.32–7.31 (m, 2H), 4.42 (t, *J* = 6.6 Hz, 1H), 3.43 (ddd, *J* = 35.2, 15.6, 6.7 Hz, 2H), 2.28 (s, 3H). ^13^C-NMR (151 MHz, D_2_O) δ 173.86, 171.21, 170.58, 139.88, 133.06, 133.02, 129.42, 127.34, 123.93, 119.69, 52.13, 29.60, 20.35; ESI^+^-MS: 263.0857 (calculated: 263.0849 for C_13_H_14_N_2_O_2_S [M + H]^+^); *m*/*z* (%): 277 (100), 263 (4.6, [M + H]^+^), 217 (0.8).

*2-Amino-3-(2-p-tolylthiazol-4-yl)propanoic acid* (*rac**-*****6c**): Yield: 92%; white powder; m.p. 180–190 °C for the hydrochloride salt, respectively m.p. 280–286 °C with decomposition for the free amino acid; ^1^H-NMR (600 MHz, D_2_O) δ 7.72 (d, *J* = 8.1 Hz, 2H), 7.69 (s, 1H), 7.34 (d, *J* = 8.1 Hz, 2H), 4.45 (t, *J* = 6.9 Hz, 1H), 3.50 (ddd, *J* = 22.8, 16.5, 6.9 Hz, 2H), 2.32 (s, 3H). ^13^C-NMR (151 MHz, D_2_O) δ 172.29, 170.18, 145.07, 143.56, 130.38, 127.38, 124.49, 120.25, 51.82, 28.80, 20.78; ESI^+^-MS: 263.0855 (calculated: 263.0849 for C_13_H_14_N_2_O_2_S [M + H]^+^); *m*/*z* (%): 277 (100), 263 (3.0, [M + H]^+^), 217 (0.9).

*2-Amino-3-(2-(4-chlorophenyl)thiazol-4-yl)propanoic acid* (*rac**-*****6d**): Yield: 93%; white powder; m.p. 250–260 °C for the hydrochloride salt, respectively m.p. over 300 °C with decomposition for the free amino acid; ^1^H-NMR (600 MHz, Methanol-*d*_4_) δ 7.99 (d, *J* = 8.5 Hz, 2H), 7.50 (d, *J* = 8.5 Hz, 2H), 7.46 (s, 1H), 4.45 (t, *J* = 5.8 Hz, 1H), 3.46 (ddd, *J* = 22.9, 15.5, 5.9 Hz, 2H). ^13^C-NMR (151 MHz, Methanol-*d*_4_) δ 170.97, 169.18, 151.97, 137.46, 133.08, 130.34, 129.08, 118.91, 53.62, 32.26; ESI^+^-MS: 283.0306 (calculated: 283.0303 for C_12_H_11_ClN_2_O_2_S [M + H]^+^); *m*/*z* (%): 307 (4.1, [M + Na]^+^, ^37^Cl), 305 (44.6, [M + Na]^+^, ^35^Cl), 286 (4.6, [M + 2H]^+^, ^37^Cl), 285 (36.4, [M + H]^+^, ^37^Cl), 284 (12.9, [M + 2H]^+^, ^35^Cl), 283 (100, [M + H]^+^, ^35^Cl), 277 (47.6), 263 (6.6), 256 (4.3).

### 3.4. Small Scale Enzymatic Reactions

#### 3.4.1. Lipase Screening for the Enzymatic Ring Opening of Oxazol-5(4*H*)-one *rac*-**5a**

To a solution of racemic 4-[(2-phenylthiazol-4-yl)methyl]-2-methyloxazol-5(4*H*)-one *rac*-**5a** (6 mg) in different alcohols (methanol, ethanol, *n*-propanol, *n*-butanol) (0.6 mL), different lipases (20 mg) were added. The reaction mixture was shaken at 1200 rpm at room temperature for 4.5 h. Samples were taken from the reaction mixture (20 μL), diluted to 1000 μL with a mixture of *n*-hexane and 2-propanol (4:1 *v*/*v*), filtered, and analysed by HPLC using a chiralpak IC column and a mixture of *n*-hexane and 2-propanol 80:20 (*v*/*v*) as eluent.

#### 3.4.2. CaL-B Mediated DKR of Oxazol-5(4*H*)-one *rac*-**5a** with Different Alcohols and in Different Solvents

To a solution of racemic 4-[(2-phenylthiazol-4-yl)methyl]-2-methyloxazol-5(4*H*)-one *rac*-**5a** (10 mg) in different solvents (600 μL), CaL-B (10 mg), and ethanol (3 eq.) were added. The enzymatic reactions were performed with and/or without adding 6 mg of racemisation catalyst *N*,*N*-diethylaminoethanol immobilized on single-walled carbon nanotubes. The enzymatic reactions in acetonitrile were performed using different alcohols (methanol, ethanol, *n*-propanol, and *n*-butanol) (3 eq.). The reaction mixtures were shaken at 1200 rpm at room temperature. Samples were taken from the reaction mixture (50 μL), diluted to 1000 μL with a mixture of *n*-hexane and 2-propanol (4:1 *v*/*v*), filtered, and analysed by HPLC by the same procedure as described in Section 3.4.1. The enzymatic DKR of racemic oxazolones *rac*-**5b**–**d** were performed by a similar procedure, in the presence of the racemisation catalyst.

#### 3.4.3. Enzymatic Hydrolysis of Racemic 2-Acetamido-3-(2-arylthiazol-4-yl)propanoic Acids *rac*-**3a**–**d**

To a suspension of *rac***-3a**–**d** (50 mg) in demineralized water (6 mL), adjusted to pH 8 with a solution of LiOH 1.25 M, a catalytic amount of CoCl_2_·6H_2_O (1 mg) and Acylase I (20 mg) were added. The reaction mixture was stirred at 37 °C. The enzymatic reactions were monitored by TLC using a mixture of *n*-butanol:acetic acid:water 3:1:1 (*v*/*v*/*v*). For chiral HPLC analysis, samples were taken from the reaction mixture (100 μL), diluted with Tris-buffer (0.1 mM Tris HCl, pH 8), heated with active charcoal to 90 °C for 20 min, cooled, and filtered before injection. The chiral HPLC analysis was performed using a Chiralpak Zwix(+) column and a mixture of methanol (50 mM formic acid, 25 mM DEA):acetonitrile:water 49:49:2 (*v*/*v*/*v*) as eluent.

### 3.5. Large Scale Chemoenzymatic Preparation of l-2-Arylthiazol-4-yl Alanines l-***6a**–**d***

To a solution of racemic 2-acetamido-3-(2-arylthiazol-4-yl)propanoic acid *rac*-**3a**–**d** (5 mmol) in anhydrous dichloromethane (20 mL), a solution of *N*,*N*′-dicyclohexyl carbodiimide (1.24 g, 6 mmol) in anhydrous dichloromethane (8 mL) was added dropwise at 0 °C. The reaction mixture was stirred for 1 h at 0 °C. After the completion of the reaction (total conversion verified by TLC, eluent dichloromethane:acetone 9:1), the formed precipitate of dicyclohexyl urea was removed by filtration. The solvent was distilled off at reduced pressure, at room temperature. The obtained oxazol-5(4*H*)-one *rac*-**5a**–**d** was dissolved in anhydrous acetonitrile (85 mL). To the obtained solution CaL-B (1.4 g), the racemisation catalyst *N*,*N*-diethylaminoethanol immobilized on carboxyl single-wall carbon nanotubes (860 mg) and ethanol (3 eq., 15 mmol, 875 μL) in the case of *rac*-**5a**–**c**, respectively *n*-propanol (3 eq., 15 mmol, 1120 μL) in the case of *rac*-**5d** were added. The reaction mixture was stirred at 1200 rpm at room temperature and was monitored by chiral HPLC as described in Section 3.4.1. The total conversion was achieved after 2 days when *rac*-**5a**–**c** were used as substrates, and respectively after 3 days when *rac*-**5d** was used as substrate. After the completion of the enzymatic alcoholysis of *rac*-**5a**–**d**, the enzyme and the catalyst were filtered off and were washed three times with acetonitrile and chloroform, for the complete recovery of the reaction products. The solvent was removed under reduced pressure and the crude product was purified by column chromatography, using a mixture of dichloromethane: acetone 9:1 (*v*/*v*) as eluent, affording the 2-acetamido-3-(2-arylthiazol-4-yl)propanoic esters l*-***4a**–**d** in 93%–96% yields and 78%–80% *ee* (Table 6).

The obtained l-2-acetamido-3-(2-arylthiazol-4-yl)propanoic esters l-**4a**–**d** were treated with a solution of sodium carbonate (0.53 g, 5 mmol) in water (10 mL). The reaction mixture was gently refluxed for 2 h, followed by acidifying with concentrated HCl (pH 3) and evaporation of water under reduced pressure. The obtained solid was redissolved in water (8 mL) by adjusting the pH to 8, using a solution of LiOH 1.25 M. Further Acylase I (60 mg) and CoCl_2_·6H_2_O (10 mg catalytic amount) were added and the reaction mixture was stirred at 37 °C, keeping the pH 7–8 with LiOH 1.25 M solution. The reaction was monitored by HPLC as described in Section 3.4.3. After the completion of the enzymatic hydrolysis (2 days), the reaction mixture was treated with phosphoric acid 5% until acidic pH (pH 1.5) and the enzyme was removed by centrifugation. The aqueous phase was applied to a DOWEX 50X8 cation exchange resin column. The enantiopure l-2-arylthiazol-4-yl alanines l**-6a**–**d** eluted with 2M NH_4_OH solution.

### 3.6. Immobilisation of N,N-Diethylaminoethanol on Single-Walled Carbon Nanotubes (SWCNT)

One gram of carboxyl-functionalized single-walled carbon nanotubes (SWCNT_COOH_) were suspended in anhydrous dichloromethane (12 mL) and treated with 1,1′-carbonyldiimidazole (120 mg). The mixture was sonicated for 5 min, and then shaken for 8 h at 1300 rpm. The suspension was filtered under reduced pressure and the precipitate was washed with anhydrous dichloromethane. The obtained filtrate was resuspended in anhydrous dichloromethane (12 mL) treated with glycerol diglycidyl ether (2 mL) and shaken for 24 h. The precipitate was then filtered under reduced pressure and washed several times with water. The obtained derivatized SWCNTs were suspended in anhydrous dichloromethane and treated with *N*,*N*-diethylaminoethanol (4 mL of anhydrous dichloromethane and 400 μL of *N*,*N*-diethylaminoethanol for 300 mg of derivatized SWCNTs). The mixture was shaken for 24 h and then filtered. The obtained precipitate was washed several times with anhydrous dichloromethane, dried, and used in the enzymatic DKR process.

## 4. Conclusions

An efficient chemoenzymatic procedure for the synthesis of various enantiopure l-2-arylthiazol-4-yl alanines was developed, based on the DKR of the corresponding oxazolones *rac*-**5a**–**d**. The novel SWCNT-immobilized amino functionalities proved to be efficient and mild racemisation agents in the CaL-B catalysed DKR process involving the stereoselective ring opening of oxazol-5(4*H*)-ones in organic media, not affecting the enzyme selectivity and activity, thus affording the corresponding N- and C- protected l-amino acids (78%–80% *ee*) with 93%–96% yields. In the next steps, the chemical hydrolysis at the ester function in mild basic conditions followed by an enantioselective hydrolysis of the amide bond, mediated by Acylase I, in aqueous media, yields final products with *ee* values increased to more than 99%, due to the l-specificity of Acylase I. The developed chemoenzymatic DKR-KR procedure was optimized and successfully applied for the preparative production of enantiopure l-2-arylthiazol-4-yl alanines, with 74%–78% global yields.
